# Suspension Array for Multiplex Detection of Eight Fungicide-Resistance Related Alleles in *Botrytis cinerea*

**DOI:** 10.3389/fmicb.2016.01482

**Published:** 2016-09-21

**Authors:** Xin Zhang, Fei Xie, Baobei Lv, Pengxiang Zhao, Xuemei Ma

**Affiliations:** College of Life Science and Bioengineering, Beijing University of TechnologyBeijing, China

**Keywords:** *Botrytis cinerea*, fungicide resistance, multiplex detection, suspension array, ASPE PCR

## Abstract

A simple and high-throughput assay to detect fungicide resistance is required for large-scale monitoring of the emergence of resistant strains of *Botrytis cinerea*. Using suspension array technology performed on a Bio-Plex 200 System, we developed a single-tube allele-specific primer extension assay that can simultaneously detect eight alleles in one reaction. These eight alleles include E198 and 198A of the β-Tubulin *gene* (*BenA*), H272 and 272Y of the Succinate dehydrogenase iron–sulfur subunit gene (*SdhB)*, I365 and 365S of the putative osmosensor histidine kinase gene (*BcOS1*), and F412 and 412S of the 3-ketoreductase gene (*erg27*). This assay was first established and optimized with eight plasmid templates containing the DNA sequence variants *BenA-*E198, *BenA-*198A, *SdhB-*H272, *SdhB-*272Y, *BcOS1-*I365, *BcOS1-*365S, *erg27*-F412, and *erg27*-412S. Results indicated that none of the probes showed cross-reactivity with one another. The minimum limit of detection for these genotypes was one copy per test. Four mutant plasmids were mixed with 10 ng/μL wild-type genomic DNA in different ratios. Detection sensitivity of mutant loci was 0.45% for *BenA-*E198A, *BcOS1-*I365S, and *erg27*-F412S, and was 4.5% for *SdhB-*H272Y. A minimum quantity of 0.1 ng of genomic DNA was necessary to obtain reliable results. This is the first reported assay that can simultaneously detect mutations in *BenA*, *SdhB*, *BcOS1*, and *erg27*.

## Introduction

*Botrytis cinerea* is the causal agent of gray mold, which often causes heavy losses on many economically important crops, including vegetables, fruits, ornamentals, and bulbs. *B. cinerea* can attack leaves, stems, fruits, and even stored and transported agricultural products (Mbengue et al., 2016). It has been named one of the top 10 fungal pathogens in molecular plant pathology (Dean et al., 2012). Synthetic fungicides are widely used for controlling disease caused by *B. cinerea*. A few years after the introduction of fungicides, development of resistance in pathogen populations was observed, and *B. cinerea* was one of the first fungi for which resistance was described (Yourman et al., 2001; Hahn, 2014).

Isolates of *B. cinerea* that are resistant to benzimidazole (e.g., carbendazim, benomyl), succinate dehydrogenase inhibitors (SDHIs) (e.g., boscalid), dicarboximide (e.g., iprodione and procymidone), and sterol biosynthesis inhibitors (SBIs) (e.g., fenhexamid) are distributed worldwide (Yourman et al., 2000; Bardas et al., 2010; Leroux et al., 2010; Van der Heyden et al., 2014; Fernandez-Ortuno et al., 2015). Increasing occurrence of fungicide resistant *B. cinerea* has become a serious problem for disease control.

The most important resistance mechanism of *B. cinerea* is modification of the fungicide target caused by mutations in the encoding genes. Point mutations at codon 198 or 200 in the β-Tubulin *gene* (*BenA*) are related to benzimidazole resistance (Leroux et al., 2002). E198A mutant strains that possess amino acid replacement of glutamic acid by alanine have been detected worldwide as major benzimidazole-resistant strains in the field (Banno et al., 2008). A histidine to tyrosine replacement at codon 272 (H272Y) in Succinate dehydrogenase iron–sulfur subunit gene (*SdhB)* is responsible for the boscalid resistance phenotype (Yin et al., 2011). Resistance to dicarboximide can be associated with point mutations in the putative osmosensor histidine kinase gene (*BcOS1*), and strains possessing the I365S mutation are dominant (Cui et al., 2002; Oshima et al., 2002). A mutation at codon 412 (F412S) in the 3-ketoreductase gene (*erg27*) that causes amino acid replacement of a phenylalanine residue by serine is related to fenhexamid resistance (Debieu et al., 2013).

At present, many molecular methods have been used to detect fungicide resistance in *B. cinerea*, including sequencing (Leroux et al., 2010; Fillinger et al., 2012), polymerase chain reaction restriction fragment length polymorphism (PCR-RFLP; Ishii et al., 2009; Van der Heyden et al., 2014), allele-specific PCR (AS PCR; De Miccolis et al., 2012, 2014; Yin et al., 2012; Van der Heyden et al., 2014), tetra-primer amplification refractory mutation systems (ARMSs) PCR (Munoz et al., 2009), real-time PCR (Banno et al., 2008; Billard et al., 2012), and high-resolution melting (HRM) analysis (Chatzidimopoulos et al., 2014). However each of these molecular assays can only detect a single allele or simultaneously detect different alleles in a single gene. Simultaneous detection of multiple alleles in a single reaction vessel is a technical difficulty in fungicide resistance identification. Increasing fungicide resistance has been a serious problem in disease control. Undoubtedly, investigating the large numbers of strains resistant to different kinds of fungicides requires massive effort. Therefore, a simple and high-throughput genotyping assay is required for large scale detection and monitoring of the emergence of resistant strains of *B. cinerea*.

**FIGURE 1:**
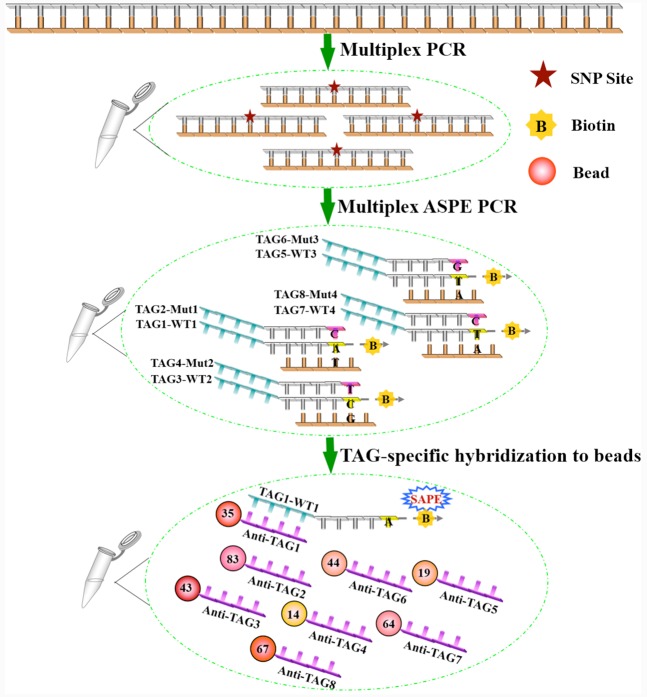
**Schematic illustration of the suspension assay.** The target regions *BenA*, *SdhB*, *BcOS1*, and *erg27* containing SNPs are amplified by 4-plex PCR in a single tube. TAG-ASPE primers overlap the SNP site in the PCR-amplified target DNA, and only perfectly matching primers can be extended (eight TAG-ASPE primers were mixed in one reaction). Biotin-labeled extended products then hybridize to the microsphere coupled with anti-TAG sequences and are detected on the Bio-Plex platform.

The Bio-Plex^®^ suspension array could be potential candidate for developing such a genotyping tool. The Bio-Plex^®^ suspension array is based on the flexible multi-analyte profiling (xMAP) technology (Houser, 2012). It enables multiplex biological testing of up to 500 analytes within a single sample volume (Spierings and Dunbar, 2013). These 500 analytes are realized by 500 distinctly colored bead sets created by the use of three fluorescent dyes at different ratios. Each bead couples to sequence-specific capture oligos that recognize and bind target DNAs. Once bound, target DNA molecules, which are labeled with biotin, are tagged with Streptavidin-R-phycoerythrin (SAPE; Dunbar, 2006; **Figure 1**). The beads are individually analyzed by cytometry on the Bio-Plex 200 platform. A red laser distinguishes the internal bead dyes to identify each microsphere particle, and a green laser provides a quantitative result of the target molecules. Many applications for nucleic acid assay have been developed using bead-based technology, including single nucleotide polymorphism (SNP) analysis (Ozaki et al., 2012, 2014; Spierings and Dunbar, 2013; Lee et al., 2015; Thierry and Derzelle, 2015), gene expression analysis (Yang et al., 2001; Naciff et al., 2005), microRNA analysis (Wang et al., 2012; Li et al., 2014), and microbiological detection (Dunbar and Jacobson, 2007; Preuner and Lion, 2013; Glushakova et al., 2015).

This study established a novel and universal multiplex genotyping method. We describe a genotyping assay that combines an allele-specific primer extension (ASPE) with the Bio-Plex suspension array. Four mutations most commonly associated with fungicide resistance in *B. cinerea* are included in this assay, including *BenA* (E198A), *SdhB* (H272Y), *BcOS1* (I365S), and *erg27* (F412S). The development of this multiplex assay will greatly reduce the time, labor, and cost of screening compared to single-reaction-based detection methods. It represents important progress in the development of multiplex genotyping technologies for molecular detection of fungicide-resistance in *B. cinerea*.

## Materials and Methods

### Strains and Culture Conditions

The Bc05.10 strain of *B. cinerea* used as a sensitive strain was provided by Jingao Dong of the Agricultural University of Hebei (Hebei, China). Field strains resistant to different types of fungicides were provided by Chaoxi Luo of the Huazhong Agricultural University (Hubei, China). *B. cinerea* isolates were incubated on potato dextrose agar (PDA) medium at 22°C for 5 days. To extract genomic DNA, mycelial disks of *B. cinerea* isolates were cultured in potato dextrose (PD) liquid medium at 22°C for 7 days. After 7 days incubation, mycelia were harvested by filtration through sterile filter paper, and dried between two layers of filter paper. Fungal material was frozen at -80°C for later use.

### DNA Extraction and Plasmid Construction

Genomic DNA was extracted from each isolate using a previously described modification of the CTAB (Agbagwa et al., 2012). Template DNA was stored at -20°C until use. Based on the *B. cinerea* sequence of *BenA*, *SdhB*, *BcOS1*, and *erg27* in GenBank (accession numbers Z69263, AY726618, AF435964, and AY220532), eight nucleotide sequences containing the variants *BenA-*E198 (GAG), *BenA-*198A (GCG), *SdhB-*H272 (CAC), *SdhB-*272Y (TAC), *BcOS1-*I365 (ATC), *BcOS1-*365S (AGC), *erg27*-F412 (TTC), and *erg27*-412S (TCC) were cloned into pUCm-T plasmids (Shanghai Sangon, CN). Gene splicing by overlap extension PCR was used for site-directed mutagenesis. All plasmid DNA constructs were confirmed by DNA sequencing. These plasmids were used to optimize reaction conditions and determine the analytical sensitivity and specificity of the bead-based suspension array. Genomic DNA and plasmid DNA were quantified using a NanoDrop 1000 Spectrophotometer (Thermo Scientific, USA).

### Primers and TAG Sequences

All primers used in multiplex PCR and ASPE PCR were designed using the software primer premier 5. TAG sequences were designed by the Luminex Corporation. All oligonucleotides used in the assay were purified by HPLC and are listed in **Tables 1**–**3**.

**Table 1:** Primers used in multiplex PCR.

Gene	Primer name	Sequences (5′–3′)	Primer concentration (μM)	PCR product size (bp)
*BenA*	Tub-F	GTCGTCCCATCGCCAAAGGT	0.05	353
	Tub-R	ACGGTGACAGCACGGAAAGA	0.05	
*SdhB*	SDH-F	ACACCGACCCAGCACCAGA	0.4	428
	SDH-R	TTAGCAATAACCGCCCAAA	0.4	
*BcOS1*	BcOS1-F	AGGTCACCCGCGTAGCAAGA	0.2	223
	BcOS1-R	TGCTTGATTTCACCCTTACA	0.2	
*erg27*	erg27-F	GCGTGGAGAACTCTAAATCGG	0.2	191
	erg27-R	AGTGTAAGGCTTGATGGTATGC	0.2	


**Table 2:** Primers used in multiplex ASPE PCR.

Gene	Primer name	Sequences (5′–3′)
*Ben A*	TAG-EAwt-F0	CATCTTCATATCAATTCTCTTATTTTGGTTGAGAACTCTGACGA
	TAG-EA-F0	ACAATATACATCACTTAAACTTTCTTGGTTGAGAACTCTGACGC
*SdhB*	TAG-HYwt-R0	AACTTTCTCTCTCTATTCTTATTTGAGCAGTTGAGAATAGTGTG
	TAG-HY-R0	AATTTCTTCTCTTTCTTTCACAATGAGCAGTTGAGAATAGTGTA
*BcOS1*	TAG-ISwt-R0	ATACTTTACAAACAAATAACACACTGCCCTGGACGCCTTCGA
	TAG-IS-R0	TCATCACTTTCTTTACTTTACATTTGCCCTGGACGCCTTCGC
*erg27*	TAG-FSwt-R0	TTCAATTCAAATCAAACACATCATCCATCCATCTTACAAGGTAGA
	TAG-FS-R0	ATCTCAATTACAATAACACACAAACATCCATCTTACAAGGTAGG


*TAG sequences are indicated by underlining.*

**Table 3:** MagPlex-TAG microspheres.

Gene	Genotype	Beads number	Anti-TAG coupled to beads (5′–3′)
*Ben A*	E198 (GAG)	MTAG-A035	AATAAGAGAATTGATATGAAGATG
	198A (GCG)	MTAG-B083	GAAAGTTTAAGTGATGTATATTGT
*SdhB*	H272 (CAC)	MTAG-A043	AAATAAGAATAGAGAGAGAAAGTT
	272Y (TAC)	MTAG-A014	ATTGTGAAAGAAAGAGAAGAAATT
*BcOS1*	I365 (ATC)	MTAG-A019	GTGTGTTATTTGTTTGTAAAGTAT
	365S (AGC)	MTAG-A044	AATGTAAAGTAAAGAAAGTGATGA
*erg27*	F412 (TTC)	MTAG-A064	ATGATGTGTTTGATTTGAATTGAA
	412S (TCC)	MTAG-A067	TTTGTGTGTTATTGTAATTGAGAT


### Multiplex PCR (4-Plex)

Multiplexed PCR to amplify the SNP-containing target regions *BenA*, *SdhB*, *BcOS1*, and *erg27* was performed in 25 μL reaction mixtures. Reactions consisted of 1 ng genomic DNA template of the Bc05.10 isolate (or different concentrations of plasmid DNAs), 1× PCR reaction buffer (-Mg^2+^), 1.5 mM MgCl_2_, 0.2 mM dNTP (TaKaRa, Japan), 2 Units of Platinum Taq DNA polymerase (Invitrogen, Carlsbad, CA, USA), 0.05 μM of each *BenA* primer, 0.4 μM of each *SdhB* primer, and 0.2 μM of the other primers. Details of multiplexed PCR primers are listed in **Table 1**. Sterile double distilled H_2_O (ddH_2_O) was used as a negative control. PCR was conducted in a thermal cycler as follows: 5 min at 94°C and 35 cycles of 30 s at 94°C, 30 s at 54°C and 30 s at 72°C, with a final extension at 72°C for 10 min, and was kept at 4°C until use. To remove unused primers, 3 μL of ExoSAP-IT (Affymetrix, SE) was added to 7.5 μL PCR products. This mixture was incubated in a thermal cycler at 37°C for 30 min, at 80°C for 15 min, and was then held at 4°C.

### Multiplex ASPE PCR

ExoSAP-IT-treated PCR product was used as the template for the Multiplex ASPE reaction. ASPE primers were designed a way that 3′ end was complementary to the wild-type (WT) or mutant (Mut) base for WT and Mut discrimination. The Platinum *Tsp* DNA polymerase (Invitrogen, Carlsbad, CA, USA) can synthesize new DNA containing biotin labeled nucleotides when the 3′ base of the ASPE primer is completely complementary, but no product can be generated if its 3′ base is mismatched. Each ASPE primer that identified a genotype required a unique TAG sequence on its 5′ end (**Table 2**). These TAG sequences were complementary to anti-TAG sequences coupled to microspheres. Multiplex ASPE PCR was performed with all eight TAG-ASPE primers in a single tube. ASPE reaction mixtures in a final 20 μL volume consisted of 1 μL ExoSAP-IT-treated PCR product, 20 mM Tris-HCl (pH 8.4), 50 mM KCl, 1.25 mM MgCl_2_, 0.75 U of Platinum *Tsp* DNA polymerase, 25 nM of each TAG-ASPE primer, and 5 μM each of dATP, dTTP, dGTP, and μM biotin-dCTP (Invitrogen, Carlsbad, CA, USA). Reaction mixtures were incubated at 96 for 2 min and 30 cycles at 94°C for 30 s, 55°C for 1 min, and 37°C for 2 min, with a final hold at 4°C until use.

### Hybridization to MagPlex-TAG Microspheres and Detection

Approximately 2500 MagPlex-TAG microspheres (Luminex, USA) of each set per reaction were mixed in a single tube. This assay required eight microsphere sets corresponding to eight genotypes. The eight microbead types selected were 35, 83, 43, 14, 19, 44, 64, and 67 (**Table 3**). The mixture was diluted to 100 of each microsphere set per μL in 2×Tm Hybridization Buffer (0.4 M NaCl, 0.2 M Tris, 0.16% Triton X-100, pH 8.0), resuspended by vortexing and sonicated for approximately 20 s. The hybridization reaction was performed in a total volume of 50 μL containing 25 μL of the MagPlex-TAG microsphere mixture, 5 μL of each ASPE reaction, and 20 μL ddH_2_O. These mixtures were denatured at 95°C for 90 s and incubated at 37°C for 1 h. After incubation, samples were filtered through 1.2 μm MultiScreen^® _HTS_^-BV 96-well filtration plates (Merck Millipore, DE) and washed twice with 75 μL of 1× Tm Hybridization Buffer (0.2 M NaCl, 0.1 M Tris, 0.08% Triton X-100, pH 8.0). Microspheres were then resuspended in 120 μL of 1× Tm Hybridization Buffer containing 0.1% BSA and 2 μg/mL SAPE (Invitrogen, USA), transferred to new 96-well plates (Corning, NY, USA) and incubated at 37°C for 15 min. Samples at 50 μL were then analyzed at room temperature on the Bio-Plex 200 System (Bio-Rad, USA). For each sample, the median fluorescence intensity (MFI) of at least 100 beads was analyzed for each bead set.

### Limits of Detection

To determine the limits of detection for each genotype, each plasmid was diluted to 10^4^, 10^3^, 10^2^, 10, and 1 copies/uL and was tested with the multiplex suspension array. To determine the lowest concentration of genomic DNA of field isolates needed in the suspension assay, genomic DNA of the IR-2 strain (benzimidazole-resistant strain) was diluted to 10, 1, 0.1, and 0.01 ng/μL. One microliter was then used as the template for multiplex PCR. The detection limit and the lowest concentration of genomic DNA were defined as the values where all replicates gave positive results for each allele. All assays were performed three times.

### Determination of Sensitivity

To determine sensitivity for each Mut allele, we mixed 10, 10^2^, 10^3^, 10^4^, and 10^5^ copies of Mut plasmid DNA with 10 ng genomic DNA (equivalent to 2.2 × 10^5^ genomic DNA copies) as the template for 4-Plex PCR. These correspond to rates of 0.045, 0.45, 4.5, 45, and 450%. The lowest rate at which all three replicates were positive was defined as the sensitivity of the assay.

### Data Analysis

For each sample tested with the Bio-Plex 200 system, MFI values were collected for each of the eight microsphere sets corresponding to each allele. Results can be exported from the Bio-Plex Manager 4.0 software into an Excel file. For each allele, NET MFI values were obtained by subtracting the respective no-target (multiplex PCR negative control) MFI values. A criterion was set as the mean MFI of 22 blank controls +5 standard deviation (SD). MFI values for each allele were required to meet this criterion, and alleles could then be determined based on allelic ratios (AR) as follows:

$$\mathrm{Mutant}\,\;allelic\,\;ratio=NET\,\;{MFI}_{Mut}/\left(NET\,\;{MFI}_{WT}+NET\,\;{MFI}_{Mut}\right)$$

Based on preset criteria, AR was used to discriminate WT, mutant, and heterozygous samples. In this study, a mutant AR ≥ 0.03 was set to call the presence of *BenA-*198A, AR ≥ 0.2 was set to call the presence of *SdhB-*272Y, AR ≥ 0.02 was set to call the presence of *BcOS1-*365S, and AR ≥ 0.06 was set to call the presence of *erg27*-412S. When the AR was lower than these values, the WT was called. All data were analyzed and plotted using Excel 2013 or GraphPad prism 5.01.

### Application to Field Isolates of *B. cinerea*

DNA samples from 10 field isolates of *B. cinerea* were analyzed using the Bio-Plex suspension array. One nanogram of genomic DNA was used as the template for multiplex PCR. To evaluate accuracy, genotyping results were compared with the results obtained by sequencing (Sangon, China).

## Results

### Multiplex PCR (4-Plex)

Multiplexed PCR of genomic DNA was performed under optimized conditions. The PCR products contained mutations of interest. Four expected PCR products were amplified in a single tube: 353 bp for *BenA*, 428 bp for *SdhB*, 223 bp for *BcOS1*, and 191 bp for *erg27*.

### Specificity of Bio-Plex Suspension Array

**FIGURE 2:**
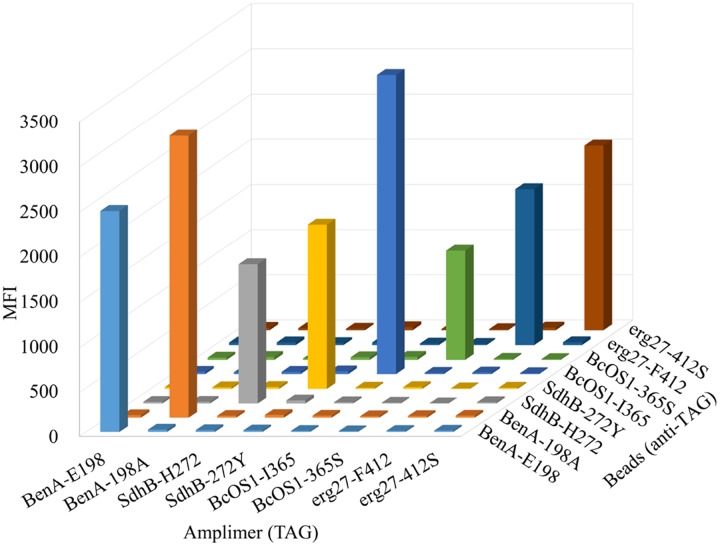
**Specificity of bead-based assay.** Individual plasmids (1 × 10^4^ copies/test) representative of each of the eight alleles were used as the template in a 4-plex PCR to determine specificity. Results showed that median fluorescence intensity (MFI) values were all above 1200. The data shown are the means of three replicates.

The specificity of the assay was verified with amplimers of each individual plasmid. Then, PCR products were hybridized to all eight sets of microbeads. As shown in **Figure 2**, no cross-reactivity occurred between each probe. The mean background MFI for each allele ranged from 12 to 27, and the signal-to-noise ratio (positive MFI/ background) ranged from 81 to 137. Thus, the genotyping assay for these eight alleles was highly specific.

### Detection Limit

**FIGURE 3:**
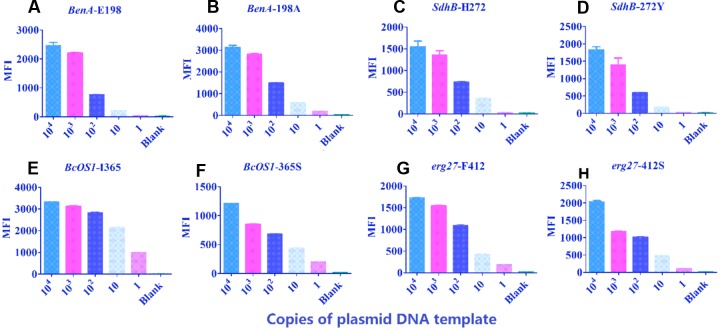
**Detection limit of the assay.** Ten-fold serial dilutions of the eight plasmids were tested with the suspension assay to determinate the limit of detection. **(A)** *BenA*-E198; **(B)** *BenA*-198A; **(C)** *SdhB*-H272; **(D)** *SdhB*-272Y; **(E)** *BcOS1*-I365; **(F)** *BcOS1*-365S; **(G)** *erg*-F412; **(H)** *erg*-412S. The data shown are the means plus standard deviation (SD) (error bars) (*n* = 3).

A series of 10-fold dilutions of each plasmid (1–10^4^ copies/μL) were used to determine the limits of detection for this method. The results indicated that MFI values reached threshold values when DNA concentrations of *BenA-*E198 (GAG), *SdhB-*H272 (CAC), *SdhB-*272Y (TAC), *erg27*-F412 (TTC), and *erg27*-412S (TCC) plasmids were as low as 10 copies. MFI values reached threshold values when DNA concentrations of *BenA-*198A (GCG), *BcOS1-*I365 (ATC), and *BcOS1-*365S (AGC) plasmids were as low as 1 copy (**Figure 3**).

**FIGURE 4:**
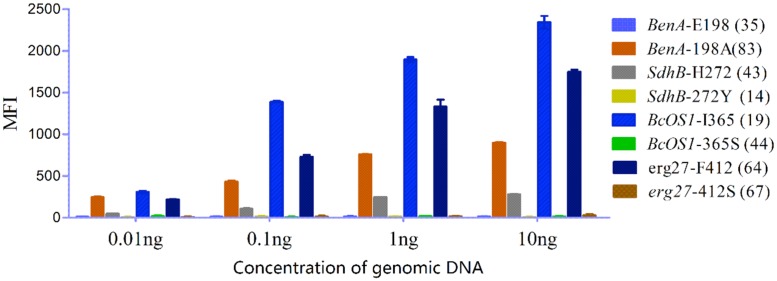
**Detection limit of genomic DNA.** Ten-fold serial dilutions of the field isolate of *Botrytis cinerea* genomic DNA template were tested with the suspension assay. The data shown are the means plus SD (*n* = 3).

Genomic DNA of field isolate strains was serially diluted (0.01–10 ng/μL) to determine the lowest DNA concentration needed in the assay. Results showed that more than 0.1 ng of input DNA was required for discrimination of all eight alleles (**Figure 4**).

### Assay Sensitivity

**FIGURE 5:**
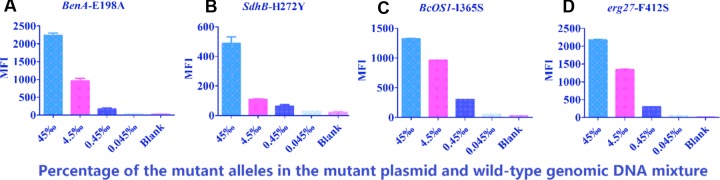
**Detection sensitivity of fungicide-resistance mutations at four sites.** Ten-fold serial dilutions of the Mut plasmid template against the WT genomic DNA template were tested with the suspension assay. **(A)** *BenA*-198A plasmid mixed with WT genomic DNA**. (B)** *SdhB*-272Y plasmid mixed with WT genomic DNA. **(C)** *BcOS1*-365S plasmid mixed with WT genomic DNA. **(D)** *erg*-412S plasmid mixed with WT genomic DNA. The data shown are the means plus SD (*n* = 3).

Serial dilutions of Mut plasmid templates (10–10^4^ copies) were mixed with 10 ng WT genomic DNA (corresponding to 2.2 × 10^5^ genomic DNA copies per reaction), as shown in **Figure 5**, and a significant concentration-dependent relationship was observed for each Mut allele. The suspension assay could detect 0.45% of *BenA*-198A, *BcOS1*-365S and *erg27*-412S, and detected 4.5% of *SdhB*-272Y when these mutations were mixed within the genomic DNA background. These results indicate that the beads-based array had a high sensitivity.

### Assay Reproducibility

To assess reproducibility of the assay, three independent assays were performed on different days. In each reaction, individual plasmid DNA aliquots (1 ng/μL) were used as templates for 4-plex PCR. The measurement of each sample was performed three times, and the mean MFI values were calculated. Results of reproducibility assays are shown in **Table 4**. The coefficient of variation (CV) for each probe was below 10%, indicating high reproducibility.

**Table 4:** Reproducibility results.

Alleles	Mean MFI of each independent assay	Mean MFI of three independent assays	CV (%) of three independent assays
*BenA-*E198	3210 3415 3120	3248	4.7
*BenA-*198A	3884 3989 3924	3932	1.3
*SdhB*-H272	3107 3100 3167	3125	1.2
*SdhB*-272Y	3378 3405 3489	3424	1.7
*BcOS1*-I365	6793 6887 6624	6768	2.0
*BcOS1*-365S	2351 2572 2302	2408	6.0
*erg27*-F412	4527 4626 4630	4594	1.3
*erg27*-412S	5558 5639 5488	5562	1.4


*The data shown are the means of three replicates.*

### Sample Analysis

Ten field strains of *B. cinerea* were genotyped using the Bio-Plex suspension array. The measurement of each sample was performed twice, and results are shown in **Table 5**. Some alleles at one locus were found to be neither WT nor Mut using this method, illustrating that other mutations exist. To identify the accuracy of the assay, sequencing of each sample was performed. Sequencing results corresponded to the suspension array (**Table 6**). Alleles that could not be detected using the beads-based assay correspond to other mutations. For instance, the *BenA* gene in sample 1 was neither WT nor E198A, but rather contained the E198V mutation. These results indicate that this high-throughput platform can simultaneously detect eight alleles within a single sample volume. The results from this method are clear, intuitive, and highly accurate.

**Table 5:** Median fluorescence intensity (MFI) values of 10 field isolates.

Sample no.	*BenA-*E198	*BenA-*198A	*SdhB*-H272	*SdhB*-272Y	*BcOS1*-I365	*BcOS1*-365S	*erg27*-F412	*erg27*-412S
1	13	30	**241**	20	11	**134**	**812**	23


2	24	30	**209**	22	**996**	22	**974**	26


3	16	**278**	18	10	18	13	**646**	26


4	14	**446**	18	17	18	23	**955**	19


5	16	17	**128**	17	**608**	22	**889**	16
6	14	29	**136**	18	**713**	20	**731**	23
7	**131**	26	**99**	19	**1062**	18	**870**	22
8	**346**	33	**111**	14	**1840**	36	**1146**	29
9	18	25	**183**	14	19	**148**	**836**	29
10	15	**332**	**199**	22	**877**	21	**879**	22
ddH_2_O	16	25	17	13	16	19	17	14


*Positive MFI values are the means of three replicates and are highlighted in bold.*

**Table 6:** Comparison of Bio-Plex suspension array and sequencing results.

Samples no.	Bio-Plex suspension array				Sequencing			
		
	*BenA*	*SdhB*	*BcOS1*	*erg27*	*BenA*	*SdhB*	*BcOS1*	*erg27*
1	#	WT	I365S	WT	E198V	WT	I365S	WT
2	#	WT	WT	WT	E198V	WT	WT	WT
3	E198A	#	#	WT	E198A	H272R	I365N	WT
4	E198A	#	#	WT	E198A	H272R	I365N	WT
5	#	WT	WT	WT	E198K	WT	WT	WT
6	#	WT	WT	WT	E198V	WT	WT	WT
7	WT	WT	WT	WT	WT	WT	WT	WT
8	WT	WT	WT	WT	WT	WT	WT	WT
9	#	WT	I365S	WT	E198V	WT	I365S	WT
10	E198A	WT	WT	WT	E198A	WT	WT	WT


*# indicates no calls.*

## Discussion

*Botrytis cinerea* is a widespread fungus that causes gray mold rot in many plants. Due to the different fungicide-resistant molecular mechanisms, investigating the large numbers of resistant strains in the field will require a massive amount of work. However, none of the preexisting molecular methods meet the high-throughput demands for detection presently required. Here, we successfully developed and validated a multiplex microbead-based suspension array for simultaneous detection of eight alleles using the Bio-Plex 200 platform. This method will be broadly applicable in the field of plant pathology.

The microbead-based suspension array combines advanced fluidics, optics, and digital signal processing. The platform provides a sensitive and ideal method for genotyping large numbers of samples at a relatively low cost. We successfully developed and validated a consolidated microbead-based genotyping platform for simultaneous detection of eight alleles in a single reaction. Four basic steps were included in this assay: (i) multiplex PCR, (ii) single-tube ASPE PCR with tagged primers, (iii) hybridization of single-stranded PCR products to microbeads carrying anti-TAG sequence, and (iv) detection on the Bio-Plex 200 platform.

With respect to the first step, we designed several primers for each gene. Different primer mixtures for the four genes were used to screen the optimized combination to ensure specificity of amplification. We also found that the 4-plex primers influenced the MFI of negative control samples. Some unsuitable primers resulted in non-specific hybridization with microbeads. After PCR primers were determined, a multiplex PCR assay was optimized for several factors, including annealing temperature, and concentrations of four primer pairs. Gel imaging illustrates the high specificity of the PCR primers used in the multiplex reaction under optimized conditions (data not shown). Because multiplex PCR was the first step of the high throughput platform, it is essential that this reaction be as specific as possible to minimize potential problems.

Multiplex ASPE PCR incorporated eight allele-specific primers to specifically target WT or mutant sites. Each 5′ end of the ASPE primer attached to a unique TAG sequence. The 24-base TAG oligonucleotides only contain three kinds of bases and exclude guanine (G). xTAG technology ensures the same annealing temperature and hybridization efficiency, so that cross hybridization between targets and microbeads can be effectively avoided. Artificial changes of nucleotides were generally introduced into the 3′ ends of ASPE primers to improve specificity. A series of allele-specific primers have been screened by real-time PCR to identify the eight alleles in our laboratory, and each primer introduced additional mutations. However, signal values that were too low were observed in suspension assays when using the same primers as used for real-time PCR. These results indicate that ASPE primers that introduced additional mutations were not suitable for this experiment. Later PCR cycles generated only single-stranded biotinylated products. This greatly improved the sensitivity of the assay and allowed for simple optimization.

Biotinylated ASPE PCR amplicons with TAG sequences were hybridized to complementary probes (anti-TAG). The eight complementary probes were each coupled to a unique color-coded microsphere. Hybridization temperature affects signal output, so 54 and 37°C were selected to identify the optimal temperature in this assay. When the temperature was 54°C, the MFI of the blank was not affected. However, the MFI of targets was lower than when the hybridization temperature was 37°C. We also found that using 1× Tm buffer containing 0.1% BSA to dilute SAPE can effectively reduce the background.

There were nine no calls obtained with the beads-based assay from 10 samples because the minimum signal requirements were not met (**Table 5**). Sequencing results indicated that these nine calls corresponded to other mutation types (**Table 6**) not within the scope of detection in this suspension assay. Except for the nine non-called alleles, the concordance between the beads-based assay and DNA sequencing was 100%. An advantage of the suspension assay is that the resulting data are easier to interpret. All results of the 10 samples were clear at a glance and can be obtained in a single day, whereas analysis of sequencing results requires additional time.

Although this assay requires four basic steps, it still saves time and effort compared to other traditional methods once reaction conditions are determined. The Bio-Plex platform uses 96-well plates, so 96 samples can be detected in one round of reactions. This is equivalent to an output of 96 × 8 = 768 genotype designations. If using traditional PCR to distinguish eight alleles, 768 reactions in 768 wells would be needed, which would be time-consuming and introduce more possibilities to make mistakes.

The Bio-Plex 200 platform allows for up to 100 different analytes to be measured simultaneously in a single reaction, so researchers can personalize their experiments according to different requirements. For this microbead-based method, more mutations in *BenA*, *SdhB*, *BcOS1*, and *erg27* could be detected: for instance, F200Y and E198V/K in the *BenA* gene (Yarden and Katan, 1993), P225T/F/L and H272Y/R in the *SdhB* gene (De Miccolis et al., 2014), I365R/N, Q369H/P and N373S in the *BcOS1* gene (Cui et al., 2004; Grabke et al., 2014), and F412I/V in the *erg27* gene (Fillinger et al., 2008). All of these mutations have been reported to be associated with fungicide resistance. Reaction conditions for multiplex PCR would not need to be further optimized; screening for these mutants would only require developing TAG-ASPE primers and microbeads corresponding to these other alleles. The microbead-based assay is flexible, and researchers can use it to design highly personalized experiments.

As with any technology, there are also some limitations of suspension arrays. First, the platform requires a relatively high start-up cost due to the commercial beads and instrument. Second, as with most traditional methods, it can only analyze known fungicide-resistance mutations. Third, this microbead-based assay requires two PCR amplifications, so it is a qualitative method and cannot be used for quantification.

This is the first report that demonstrates simultaneous detection of resistance to benzimidazoles, dicarboximides, SDHIs, and SBIs. The high-throughput capacity of the bead-based methods in fungicide resistance detection is unmatched. All results show that this multiplex, accurate, flexible, and sensitive assay represents a new efficient genotyping tool for fungicide resistance monitoring and administering of field strains of *B. cinerea*. The protocol presented in this study will be helpful to researchers for characterizing field strains of *B. cinerea*.

## Author Contributions

XZ wrote the paper and performed most of this work. BL participated in designing and screening of ASPE primers. FX and PZ interpreted the results and revised the paper. XM supervised the entire study. All authors reviewed the manuscript.

## Conflict of Interest Statement

The authors declare that the research was conducted in the absence of any commercial or financial relationships that could be construed as a potential conflict of interest.
